# Renal adverse reactions of tyrosine kinase inhibitors in the treatment of tumours: A Bayesian network meta-analysis

**DOI:** 10.3389/fphar.2022.1023660

**Published:** 2022-11-03

**Authors:** Ying Xiong, Qinxuan Wang, Yangyi Liu, Jingwen Wei, Xiaolei Chen

**Affiliations:** ^1^ Department of Periodical Press, West China Hospital, Sichuan University, Chengdu, China; ^2^ Chinese Evidence-based Medicine Center, West China Hospital, Sichuan University, Chengdu, China; ^3^ West China School of Medicine, Sichuan University, Chengdu, China; ^4^ Department of Nephrology, West China Hospital, Sichuan University, Chengdu, China

**Keywords:** Bayesian network meta-analysis, renal adverse reactions, tumours, tyrosine kinase inhibitors, treatment

## Abstract

**Objectives:** Tumours remain a serious threat to human life. Following rapid progress in oncology research, tyrosine kinase inhibitors have been used to treat multiple tumour types. Given the great influence of kidneys on pharmacokinetics, renal toxicities associated with TKIs have attracted attention. However, the TKIs with the lowest risks of renal impairment are unclear. In this study, we conducted a Bayesian network meta-analysis to compare the incidence of renal impairment among different TKIs in patients with tumours.

**Methods and analysis:** Six databases (PubMed, EMBASE, The Cochrane Library, Chinese National Knowledge Infrastructure, Wanfang Data, and China Biomedical Literature Database) were electronically searched from inception to 1 November 2021 to identify randomized controlled trials on the incidence of renal impairment for different TKIs in patients with tumours. Two reviewers independently screened the literature, extracted data, and assessed the risk of bias of the included studies. Then, a pairwise meta-analysis was conducted using Stata version 13, and network meta-analysis within the Bayesian framework was conducted using R software version 3.5.3 with the package “gemtc 0.8–2” recalling JAGS (version 4.3.0).

**Results:** Overall, 34 randomized controlled trials were included in this study. Although renal toxicity was common among patients receiving TKIs, the incidence and severity greatly differed among the drugs and studies. Elevated creatinine and protein levels were the most common nephrotoxic events, whereas haematuria was relatively rare. Among TKIs, nintedanib and ripretinib carried the lowest risks of renal impairment.

**Conclusion:** TKIs displayed different profiles of renal toxicity because of their different targets and underlying mechanisms. Clinicians should be aware of the risks of renal impairment to select the optimal treatment and improve patient adherence to treatment.

**Systematic Review Registration:** [www.crd.york.ac.uk/prospero/], identifier [CRD42022295853].

## 1 Introduction

Cancer is a serious threat to human life. According to the World Health Organization, malignant tumours represent the leading cause of disease burden as estimated using cause-specific disability-adjusted life years in both men and women (Mattiuzzi and Lippi, 2019). Globally, an estimated 19.3 million new cancer cases and almost 10 million cancer deaths occurred in 2020. Nearly 28.4 million new cancer cases are expected to occur in 2040, representing a 47% increase vs. the number in 2020 (Sung et al., 2021). Because of the rapid progress in oncology research, the treatment paradigm for tumours has changed dramatically. Surgery and radiotherapy remain the primary treatments for local and non-metastatic cancers, whereas anticancer drugs (chemotherapy, hormone therapies, and biological therapies) are used for metastatic cancers. Compared with traditional chemotherapy, novel targeted therapy, a type of biological therapy, has significantly improved clinical outcomes and provided the foundation for precision medicine in cancer treatment (White Al-Habeeb et al., 2016). Targeted therapies are designed to block specific biologic transduction pathways or cancer proteins involved in tumour growth and differentiation, thereby minimizing the death of normal cells and avoiding undesirable side effects (Perez-Herrero and Fernandez-Medarde, 2015).

Tyrosine kinases (TKs) comprise a family of proteins that contribute to the development of cancer. This class of proteins catalyzes the transfer of phosphate groups on ATP to the tyrosine residues of several proteins, thereby phosphorylating proteins and then transferring signals to regulate cell growth, differentiation, death, and a series of physiological and biochemical processes (Jiao et al., 2018). More than 50% of proto-oncogenes and oncogene products have TK activity, and their abnormal expression leads to tumourigenesis (Drake et al., 2014). Therefore, TKs have emerged as valuable targets for drug development in oncology research. Based on their structure, function, and localization, TKs can be categorized into receptor tyrosine kinases (RTKs), non-receptor tyrosine kinases (NRTKs), and dual-specificity kinases that can phosphorylate serine, threonine, and tyrosine residues. Agents targeting these proteins, so-called tyrosine kinase inhibitors (TKIs), comprise a class of small-molecule, orally administered drugs (Hartmann et al., 2009; Patterson et al., 2009; Roskoski, 2020). There are two types of TKIs, namely cellular TKIs that target NRTKs and receptor TKIs that target single receptors, such as EGFR, or multiple targets. For example, sunitinib targets vascular endothelial growth factor receptors 1–3 (VEGFR1–3), PDGFR, KIT, Flt3, and RET (Broekman et al., 2011). TKIs have been used to treat multiple cancer types, including lung cancer, breast cancer, pancreatic cancer, and gastrointestinal stromal tumours. Compared with conventional chemotherapy, TKIs are associated with significantly reduced treatment-associated toxicity (Wu et al., 2016; Holleman et al., 2019; Huang et al., 2020). However, only a few of these drugs are strictly selective for one target. Because of the ubiquitous physiological roles of protein kinases in the body, adverse events affecting various organs have been identified during TKI treatment (Petrelli et al., 2012; Hamnvik et al., 2015; Fujita et al., 2017). Given that kidneys play an important role in pharmacokinetics, renal toxicities associated with TKIs have attracted attention (Grothey et al., 2013; Troxell et al., 2016; Ramachandran et al., 2018). Furthermore, the high rates of chronic kidney disease and concomitant nephrotoxic drug administration make patients with cancer susceptible to renal injuries from treatment (Launay-Vacher et al., 2015; Zhao et al., 2021a). Hypertension, electrolyte disturbance, and renal impairment are the most commonly reported renal adverse events (Jhaveri et al., 2016). Because the first two events probably result from multiple causes, it is difficult to ascribe them to renal toxicities. We only focused on renal impairment, which was reflected by abnormal urinalysis results and elevated serum creatinine levels in this study. These abnormal results of laboratory tests, including proteinuria, haematuria, and acute or chronic renal insufficiency, are also the main reasons for the referral of patients with tumours to the nephrology department. According to information available at NIH PubChem and FDA.gov, more than 50 TKIs have been approved by the FDA. The incidence, patterns, and severity of renal adverse effects are inconsistent among different TKIs. Information about these events is important for clinical decision-making. However, the renal toxicities of TKIs, especially newer TKIs, are limited to data from case reports, series, or simple pairwise comparisons. There is no network evidence chain among a variety of TKIs, and the TKIs with the lowest incidence of renal impairment are unclear. Therefore, a network analysis that includes all TKIs and systematically measures their comparative kidney safety is of great necessity. This study used network meta-analysis to systematically evaluate the renal adverse reactions of patients with tumours after receiving TKI treatment and rank the incidence of nephrotoxicity of different TKIs to provide a reference for clinicians to choose anti-cancer drugs.

## 2 Methods

This study has been registered on PROSPERO (www.crd.york.ac.uk/prospero/) under the number PROSPERO CRD42022295853. The protocol followed the Preferred Reporting Items for Systematic Reviews and Meta-analyses Protocol (S1 Checklist) (Moher et al., 2015).

### 2.1 Patient and public involvement

It was not appropriate or possible to involve patients or the public in the design, conduct, reporting, or dissemination plans of our research.

### 2.2 Eligibility criteria

#### 2.2.1 Types of studies

Only randomized clinical trials (RCTs) that evaluated the renal adverse reactions of TKIs in the treatment of tumours were included. Observational, cohort, case–control, case series, qualitative, and laboratory studies were excluded. There were no limitations on the status, language, or year of publication.

#### 2.2.2 Types of participants

Patients with malignant tumours diagnosed by clinicopathology were eligible. No limitations were implemented regarding age, sex, tumour type, and course of disease.

#### 2.2.3 Types of interventions and comparator(s)/control

The experimental group was treated with at least one TKI, and the control group was treated with another (or additional) TKI, chemotherapy, or placebo (blank control). The experimental group was treated with at least one TKI combined with chemotherapy or routine treatment, and the control group was treated with another (or additional) TKI combined with the same chemotherapy or routine treatment.

#### 2.2.4 Types of outcome measures

The outcome measures were the incidence of renal adverse reactions, grade 1–2 adverse reactions, grade 3–4 adverse reactions, elevated serum creatinine (Scr) levels, proteinuria, and haematuria.

#### 2.2.5 Exclusion criteria

After multiple screenings, duplicate studies and studies for which the full text or complete data could not be obtained were excluded.

### 2.3 Data sources and search strategy

**FIGURE 1:**
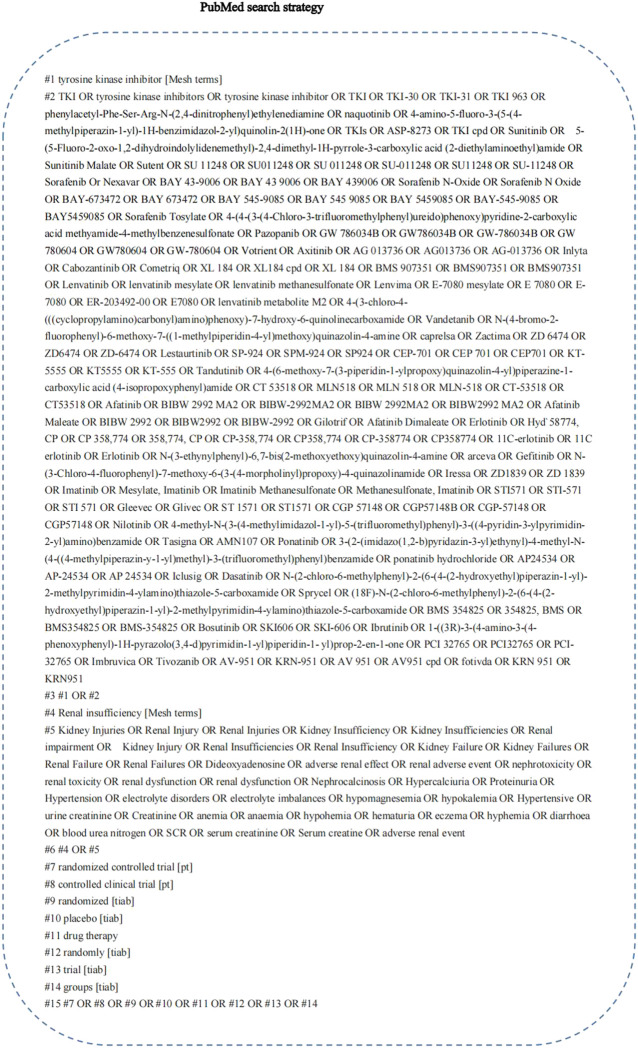
PubMed search strategy. A complete retrieval strategy of PubMed database which was used to search the literature in this study.

Six databases, namely PubMed, EMBASE, The Cochrane Library, Chinese National Knowledge Infrastructure (CNKI), Wanfang Data, and China Biomedical Literature Database, were electronically searched from inception to 1 November 2021 to identify RCTs of the incidence of renal impairment for different TKIs in patients with tumours from inception to 1 November 2021. In addition, the references of the identified studies were traced to supplement the relevant literature. Retrieval was performed using combinations of subject words and free words as follows: tyrosine kinase inhibitors, TKI, sunitinib, sorafenib, pazopanib, asitinib, cabozantinib, lenvatinib, vandetanib, lestartinib, tandutinib, afatinib, erlotinib, gefitinib, imatinib, nilotinib, ponatinib, dasatinib, bosutinib, ibrutinib, tivozanib, adverse reactions, side effects, renal injury, renal failure, elevated serum creatinine, proteinuria, haematuria, and randomization. An example of the search strategy is presented in Figure 1.

### 2.4 Literature screening and data extraction

Two evaluators independently screened the literature, extracted the data, and cross-checked the information. In case of differences, a third party was consulted to assist in judgment, and the authors of the included studies were contacted to supplement the missing data as needed. During literature screening, we first read the title and abstract and then read the full text after excluding obviously irrelevant studies to determine whether the research was suitable for inclusion. Data extraction mainly included basic information included in the study, including the research topic, first author, journal, and time of publication; the baseline characteristics of the subjects, including the number of samples in each group and the age, gender, and disease status of the patients; specific details about the intervention measures and follow-up duration key elements of bias risk assessment, and outcome indicators and outcome measurement data of concern.

### 2.5 Assessment of risk of bias

Two reviewers independently used the Cochrane Risk of Bias two tool (Sterne et al., 2019) to assess the risk of bias of each included study in terms of the following seven domains: random sequence generation (selection bias), allocation concealment (selection bias), blinding of participants and personnel (performance bias), blinding of outcome assessment (detection bias), incomplete outcome data (attrition bias), selective reporting (reporting bias), and other bias. Each domain was evaluated, and the risk of bias was graded as high, low, or unclear. The strength of the body of evidence was assessed using Grading of Recommendations Assessment, Development and Evaluation tools (Guyatt et al., 2011). The corresponding author was contacted if the data were unclear, and all disagreements were resolved by a third reviewer.

### 2.6 Statistical analysis

Conventional pairwise meta-analysis was initially performed considering the available head-to-head comparisons. We used the odds ratio (OR) or its logarithm and its 95% confidence interval (CI) to estimate the risk of renal adverse events for different regimens. A standard random-effects model was applied because of the expected variation among various regimens to provide more conservative estimated effects. Statistical heterogeneity was assessed using the I^2^ statistic (Higgins and Thompson, 2002). Bayesian network meta-analysis was conducted using random-effects generalized linear models based on the Markov chain Monte Carlo method (Gelman and Rubin, 1996). Each of the four chains was simultaneously run for 50,000 burn-ins and 100,000 inference iterations per chain to obtain the posterior distribution. The convergence of the model was detected using the Gelman–Rubin method combined with a density plot and tract plot (Brooks and Gelman, 1998). For all outcomes, we summarized the evidence by drawing a network relation graph. The renal adverse events of different treatment regimens was ranked according to the surface under the cumulative ranking (SUCRA) curve (Salanti et al., 2011). League tables were used to summarize all possible comparisons in the network, which indicated whether the estimated differences among different regimens were statistically significant. Model fit was assessed by calculating the deviance information criterion (DIC) as the sum of the posterior mean of the residual deviance and leverage pD. The transitivity assumption was evaluated by comparing the distribution of potential effect modifiers (mean age, sex ratio, sample size, and year) across treatment comparisons. In our analysis, global inconsistency was evaluated by the design-by-treatment interaction approach (Chaimani et al., 2013). To check the assumption of local consistency, the loop-specific approach and node-splitting method were used. We adopted the *τ* ^2^ test to evaluate the extent of heterogeneity for each outcome. Additionally, meta-regression and sensitivity analyses were conducted to explore the sources of heterogeneity and ensure the validity and robustness of the findings. Furthermore, to probe the rankings of all treatment regimens for the secondary outcomes, we conducted subgroup analyses based on different outcome definitions (renal adverse event grade) and cancer types. Publication bias was assessed by examining the potential presence of small study effects *via* the visual inspection of comparison-adjusted funnel plots (Dias et al., 2010). Pairwise meta-analysis was conducted using Stata version 13 (StataCorp LP, College Station, TX, USA), and network meta-analysis within the Bayesian framework was conducted using R software version 3.5.3 (R Foundation for Statistical Computing, Vienna, Austria) with the package “BUGsnet” recalling JAGS (version 4.3.0) (Neupane et al., 2014). *p* < 0.05 indicated statistical significance.

## 3 Results

### 3.1 Document screening process and results

**FIGURE 2:**
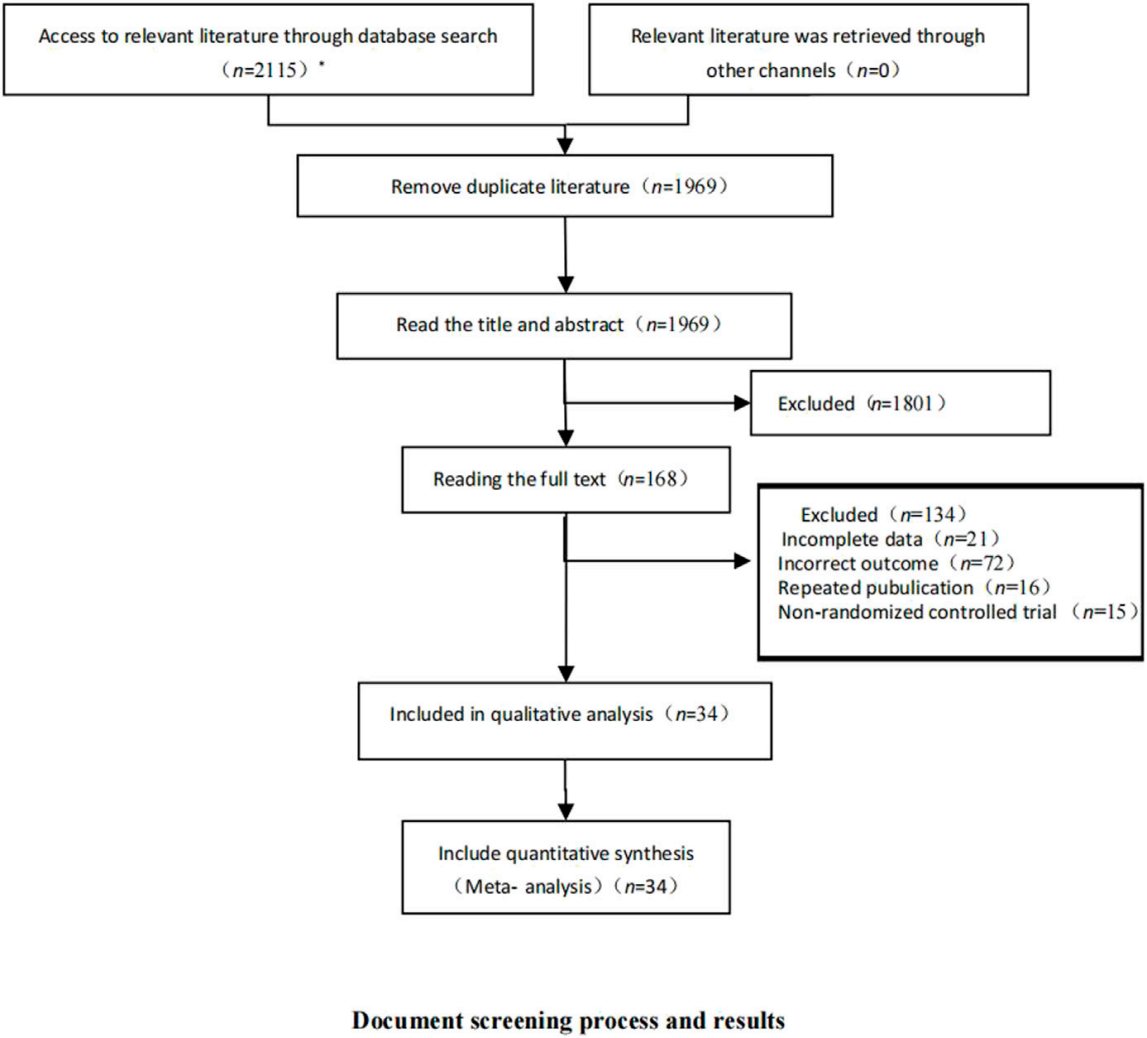
Document screening process and results. This figure shows the database and results retrieved by this study and the literature screening process. * The number of databases searched and documents checked were as follows: CNKI (*n* = 11); Wanfang Data (*n* = 45); PubMed (*n* = 998); Embase (*n* = 120); and The Cochrane Library (*n* = 941).

After database retrieval and supplementary retrieval, we obtained 2,115 documents, and after eliminating duplicate documents, we obtained 1969 documents. After reading the titles and abstracts, 168 studies were obtained. After reading the full text, 34 RCTs (Richardson et al., 2018; Zhao et al., 2021b; Motzer et al., 2013a; Motzer et al., 2013b; Sternberg et al., 2013; Rini et al., 2011; Kudo et al., 2018; Yang et al., 2017; Vilgrain et al., 2017; Spreafico et al., 2014; Sun et al., 2018; Symonds et al., 2015; Thornton et al., 2012; Qin et al., 2021; Schoffski et al., 2021; Sheng et al., 2019; Belani et al., 2014; Besse et al., 2017; Blay et al., 2020; Bukowski et al., 2007; Chi et al., 2021; Choueiri et al., 2020; Ellis et al., 2014; du Bois et al., 2014; Martin et al., 2017; Gravalos et al., 2018; Gross-Goupil et al., 2018; Zhou et al., 2019; Hall et al., 2020; Haas et al., 2021; Machiels et al., 2018; Langerbeins et al., 2015; Li et al., 2016; Schlumberger et al., 2015) were finally included (Figure 2).

### 3.2 Basic characteristics of the included studies

Most of the included RCTs were published after 2010. In total, the studies included 13,398 patients with tumours, and the tumour types were widely distributed. The studies reported 26 different interventions, including TKI therapy alone and TKI therapy combined with chemotherapy or placebo. Table 1 presents the basic characteristics of the included studies.

**TABLE 1:** Basic characteristics of the included studies.

First author	Publication year	Sample size T	Sample size C	Gender T	Gender C	Tumor type	Interventions	Control measures	Follow up time	Outcome indicators
Debra L Richardson	2018	50	50	0/50	0/50	Ovarian Cancer	paclitaxel + pazopanib	paclitaxel + placebo	17.7 months	①
Robert J. Motzer	2013	557	553	398/159	415/138	renal cell carcinoma	pazopanib	sunitinib	8 months	①②③
Cora N. Sternberg	2013	290	145	198/92	109/36	renal cell carcinoma	pazopanib	placebo	20 months	①③
Brian I Rini	2011	361	362	265/96	258/104	renal cell carcinoma	axitinib	sorafenib	20 months	①②
Masatoshi Kudo	2018	478	476	405/73	401/75	hepatocellular carcinoma	lenvatinib	sorafenib	42 months	①③
Jin-Ji Yang	2017	85	73	32/53	32/41	non-small-cell lung cancer and multiple brain metastases	Icotinib	whole-brain irradiation with or without chemotherapy	48 months	①④
Valérie Vilgrain	2017	222	237	202/20	212/25	hepatocellular carcinoma	sorafenib	selective internal radiotherapy	49 months	①②
Anna Spreafico	2014	11	11	11/0	11/0	castration-resistant prostate cancer	cediranib + dasatinib	cediranib alone	2 months	①②③④
Jong-Mu Sun	2018	48	67	40/8	43/4	Small-cell lung cancer	Pazopanib	placebo	4 months	①③
R Paul Symonds	2015	44	44	—	—	cervical cancer	cediranib	placebo	6 months	①②③
Katherine Thornton	2012	231	99	—	—	Medullary thyroid cancer	vandetanib	placebo	10 months	①②③
Shukui Qin	2021	267	133	223/38	116/16	advanced hepatocellular carcinoma	apatinib	placebo	6 months	①③
Patrick Scho¨ffski	2021	40	40	20/20	17/23	Soft tissue sarcomas	Nintedanib	Ifosfamide	3 months	①②
Robert J. Motzer	2013	260	257	185/75	189/68	metastatic renal cell carcinoma	Tivozanib	Sorafenib	5 months	①③⑤
Xinan Sheng	2019	48	24	37/11	17/7	metastatic renal cell carcinoma	axitinib	sorafenib	1 month	①③
Chandra P Belani	2014	113	57	71/42	37/20	nonsmall-cell lung cancer	Axitinib + pemetrexed/cisplatin	pemetrexed/cisplatin	2 months	①②
Besse B	2017	71	71	41/30	45/26	Lung cancer	pazopanib	placebo	2 months	①③
Jean-Yves Blay	2020	85	44	47/38	26/18	gastrointestinal stromal tumours	Ripretinib	placebo	2 months	①②
Ronald M. Bukowski	2007	51	53	33/18	40/13	metastatic renal cell carcinoma	Erlotinib + Bevacizumab	Bevacizumab	1 month	①②③
Chi, Y	2021	282	137	177/105	91/46	Colorectal cancer	anlotinib	placebo	3 months	①③④
Choueiri, T. K	2020	33	27	29/4	17/10	metastatic Papillary renal cell carcinoma	savolitinib	sunitinib	3 months	①②
Peter M Ellis	2014	480	240	244/236	120/120	non-small-cell lung cancer present with advanced or metastatic disease	dacomitinib	placebo	4 months	①②
Andreas du Bois	2014	477	461	0/477	0/461	stage II-IV epithelial ovarian, fallopian tube, or primary peritoneal carcinoma	pazopanib	placebo	T:17.9 months; C:12.3 months	①③
Miguel Martin	2017	1,420	1,420	0/1,420	0/1,420	stage 2–3c HER2-positive operable breast cancer	neratinib	placebo	5 years	②⑤
Cristina Grávalos	2018	25	24	16/9	17/7	Metastatic Colorectal Cancer	axitinib	placebo	26.07 months	①③
M. Gross-Goupil	2018	363	361	280/83	250/111	renal cell carcinoma	axitinib	placebo	NA	②③
Ai-Ping Zhou	2019	90	43	66/24	34/9	Metastatic Renal Cell Carcinoma	Anlotinib	Sunitinib	every 3 months until death or end of the study	①②
M.R. Hall	2020	59	58	0/59	0/58	advanced ovarian, fallopian tube or primary peritoneal cancer	cyclophosphamide + nintedanib	cyclophosphamide + placebo	1.6 years	①②
Hongyun Zhao	2021	157	156	66/91	62/94	Advanced EGFR-Mutant NSCLC	Apatinib + Gefitinib	Placebo + Gefitinib	15.8 months	①③④
M. Haas	2021	77	38	33/44	17/21	metastatic pancreatic cancer	Gemcitabine + afatinib	Gemcitabine	NA	①②
J.-P. Machiels	2018	23	4	16/7	1/3	squamous cell carcinoma of the head and neck	Afatinib	No treatment	NA	②
Petra Langerbeins	2015	85	85	NA	NA	Early Stage CLL	ibrutinib	placebo	NA	②
Jin Li	2016	181	92	132/44	69/22	advanced or metastatic gastric or gastroesophageal junction adenocarcinoma	apatinib	placebo	30 months	①③
Schlumberger M	2015	261	131	125/136	75/56	Radioiodine-Refractory Thyroid Cancer	lenvatinib	placebo	NA	①③

① Incidence of adverse reactions (grade 1–2 or 3–4); ② incidence of acute renal injury; ③ incidence of proteinuria; incidence of haematuria; other kidney-related adverse reactions.

NA, not available.

### 3.3 Bias risk assessment results of the included studies

**FIGURE 3:**
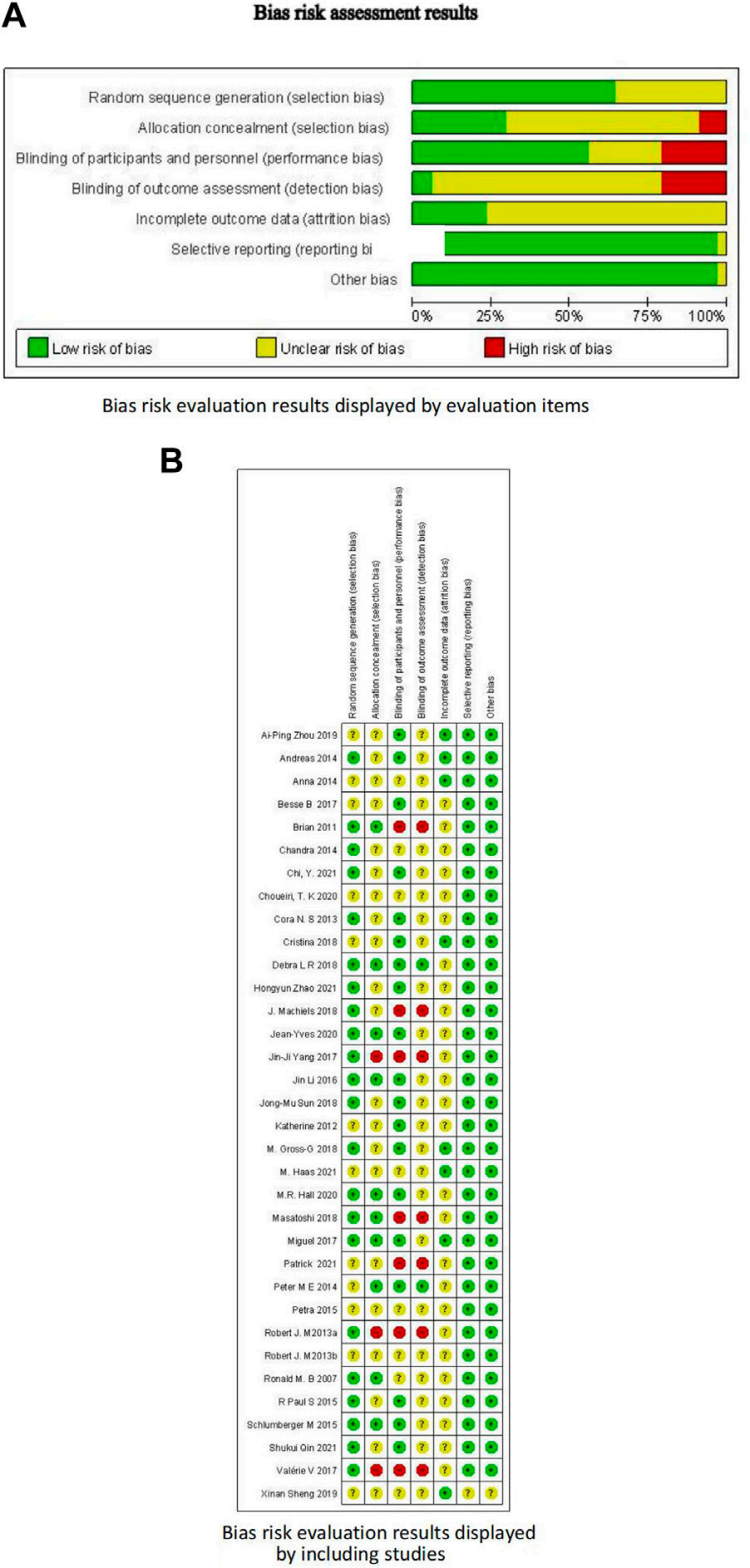
Bias risk assessment results. **(A)** Bias risk evaluation results displayed by evaluation items; **(B)** Bias risk evaluation results displayed by including studies.

Among the 34 included RCTs, 22 studies (65%) clearly reported the randomization methods, 10 studies (29%) clearly reported the allocation concealment, and 19 studies (56%) clearly reported the blinding methods. Other studies did not clearly report these methods, but they were mentioned in the article, leading to an overall low-to-moderate risk of bias. Figure 3 and Table 2 present the bias risk assessment results of the included studies.

**TABLE 2:** Results of the bias risk assessment of the included studies.

Included studies	Publication year	Random sequence generation	Blinding	Allocation concealment	Incomplete outcome data	Selective reporting	Other bias
Debra L Richardson	2018	a computer-generated random allocation sequence	double-blinded	A web-enabled centralised registration system	Lost visit	No	no
Robert J. Motzer	2013	in permuted blocks of four	open-lable	no	Lost visit, ITT	No	no
Cora N. Sternberg	2013	centrally randomly assigned	double-blind	unclear	Lost visit	No	no
Brian I Rini	2011	Randomisation lists were generated from an independent randomization group using a permuted block design of size four	no	A web-enabled centralised registration system	Lost visit	No	no
Masatoshi Kudo	2018	a computer-generated random allocation sequence	no	an interactive voice–web response system	Lost visit	No	no
Jin-Ji Yang	2017	a random web-based allocation system	open-lable	no	Lost visit	No	no
Valérie Vilgrain	2017	a computer-generated random allocation sequence	open-lable	no	Lost visit	No	no
Anna Spreafico	2014	unclear	unclear	unclear	complete	No	No
Jong-Mu Sun	2018	a random number table generated for a stratified random permuted block design	double-blinded	unclear	lost visit	No	No
R Paul Symonds	2015	a computer-generated random allocation sequence	double-blinded	unclear	lost visit	No	No
Katherine Thornton	2012	unclear	double-blinded	unclear	lost visit	No	No
Shukui Qin	2021	the randomisation sequences directly obtained from the randomisation system	double-blinded	unclear	lost visit	No	No
Patrick Scho¨ffski	2021	unclear	open-label	unclear	lost visit	No	No
Robert J. Motzer	2013	unclear	unclear	unclear	lost visit	No	No
Xinan Sheng	2019	unclear	unclear	unclear	complete	No	No
Chandra P Belani	2014	a centralized, randomized permuted block allocation	unclear	unclear	lost visit	No	No
Besse B	2017	unclear	double blind	unclear	lost visit	No	No
Jean-Yves Blay	2020	computer-generated randomization system	double blind	an interactive response technology system	lost visit	No	No
Ronald M. Bukowski	2007	computer-generated randomization system	unclear	an interactive voice response service	lost visit	No	No
Chi, Y	2021	computer-generated randomization system	double blind	unclear	lost visit	No	No
Choueiri, T. K	2020	unclear	unclear	unclear	lost visit	No	No
Peter M Ellis	2014	unclear	four-blind	unclear	lost visit	No	No
Andreas du Bois	2014	Randomassignment was stratified by first-line treatment outcome and geographic region	double-blind	unclear	lost visit/ITT analysis	no	No
Miguel Martin	2017	The randomisation sequence was generated *via* permuted blocks	double-blind	an interactive voice and web-response system	lost visit/ITT analysis	no	No
Cristina Grávalos	2018	unclear	double-blind	unclear	complete/ITT analysis	no	No
M. Gross-Goupil	2018	randomassignment was stratified by country and/or risk group	double-blind	unclear	lost visit/ITT analysis	no	No
Ai-Ping Zhou	2019	unclear	double-blind	unclear	complete	no	No
M.R. Hall	2020	stratified randomisation according to age, previous lines of chemotherapy	double-blind	an interactive webbased system	lost visit	no	No
Hongyun Zhao	2021	Randomization was done by minimization using BioKnow—randomization	double-blind	unclear	lost visit	no	No
M. Haas	2021	unclear	unclear	unclear	lost visit/ITT analysis	no	No
J.P. Machiels	2018	Randomization was stratified according to the patient institution	no	unclear	Lost visit	no	No
Petra Langerbeins	2015	unclear	unclear	unclear	Lost visit	no	No
Jin Li	2016	computer-generated randomization schedule	double-blind	an interactive Web-response or voice-response system	Lost visit	no	No
Schlumberger M	2015	Block randomization was performed by an interactive voice-response and Web-response system	double-blind	an interactive voice-response and Web-response system	yes	no	No

### 3.4 Results of network meta-analysis

#### 3.4.1 Network diagram

After using the data. prep () function to prepare the data, we used the net. plot () function to describe the research network graphically. The net. plot () function can output the network diagram of outcome indicators as required, the size of nodes is proportional to the number of studies included in each group, and the thickness of node connections is proportional to the number of studies directly compared at both ends of the nodes. If a closed loop is formed between nodes, this indicates that these studies can be simultaneously included in the comparison.

**FIGURE 4:**
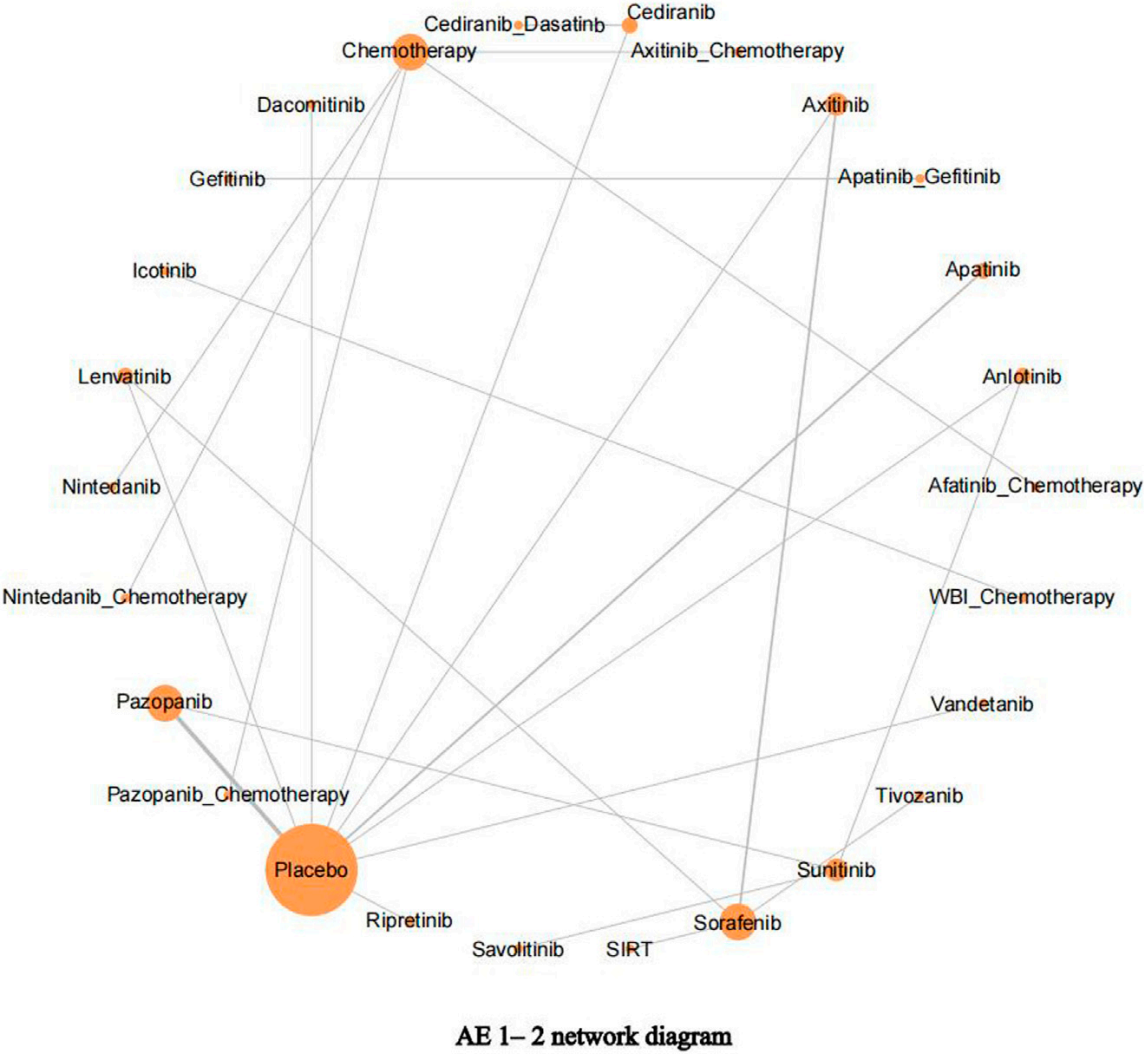
Adverse events (AE) 1–2 network diagram. The network diagram of outcome indicators AE 1–2, the size of nodes is proportional to the number of studies, and the thickness of node connections is proportional to the number of studies directly compared at both ends of the nodes.

Using adverse events (AE) 1–2 as an example, it is apparent from Figure 4 that in addition to icotinib and WBI–chemotherapy, the other 24 interventions could form a closed loop. Among them, pazopanib, apatinib, and placebo have been more extensively studied, followed by lenvatinib, axitinib, and sorafenib.

#### 3.4.2 Heterogeneity and consistency test

**FIGURE 5:**
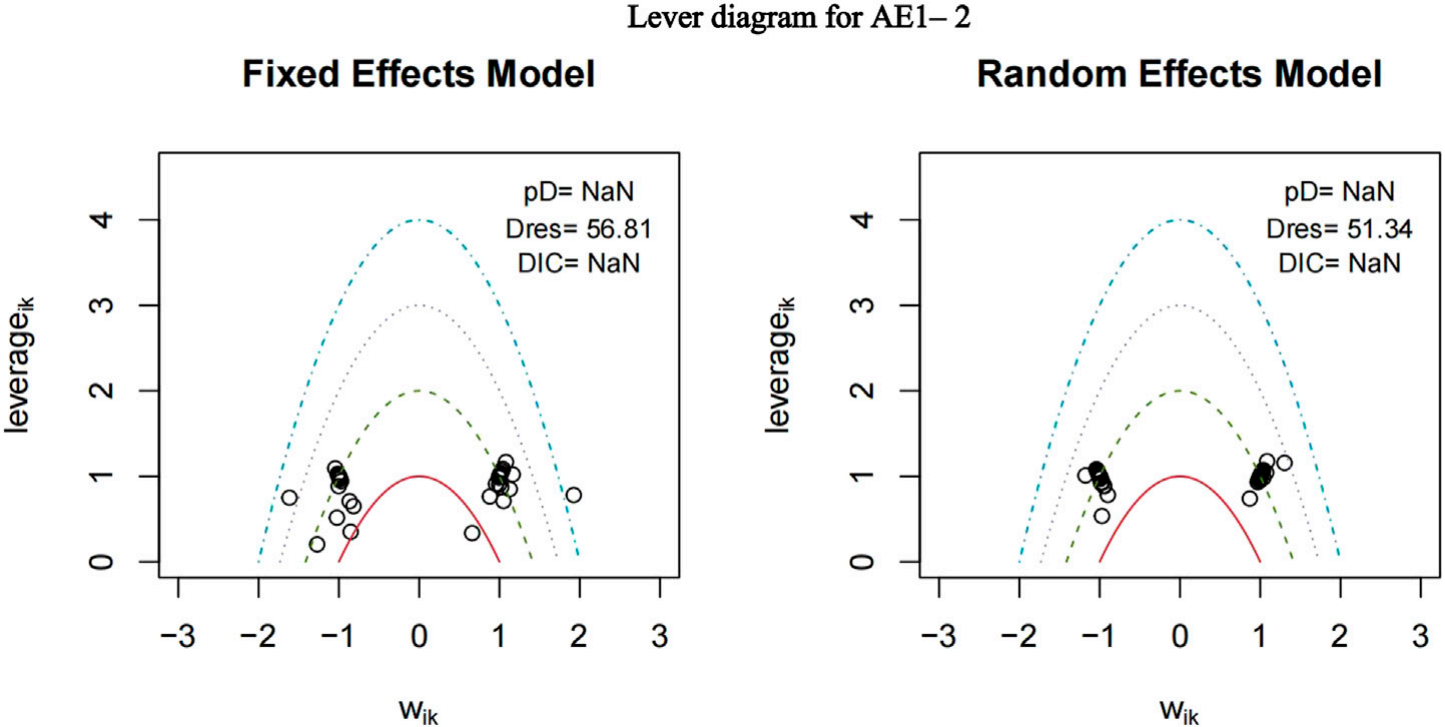
Lever diagram for adverse events (AE) 1–2. The lever diagram presents the comparison between leverageik and Bayesian deviation residuals of all I tests and each of the K arms.

**FIGURE 6:**
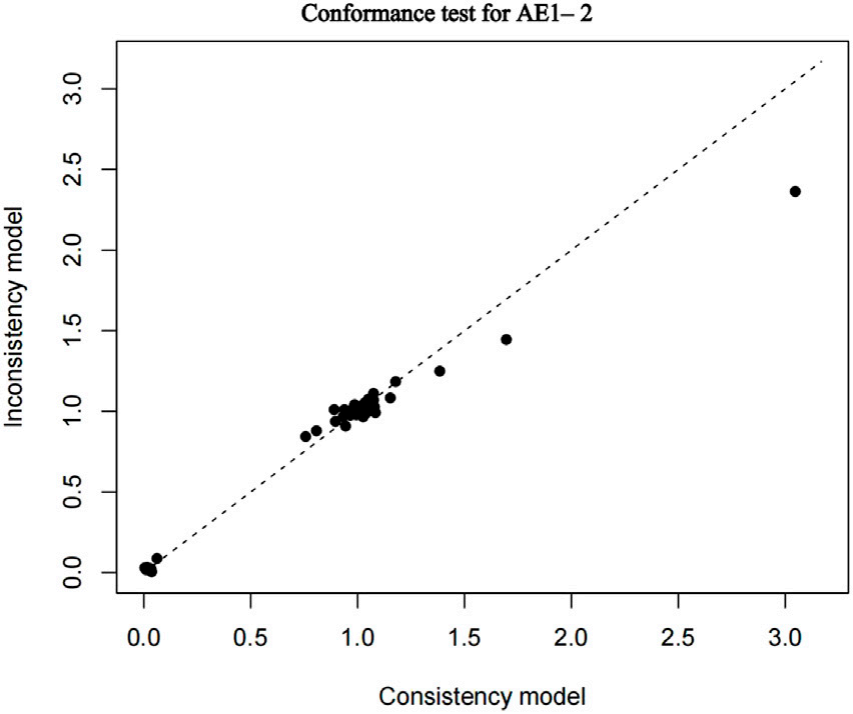
Conformance test for adverse events (AE) 1–2. Conformance test compares the posterior mean deviation of each data group between consistency and the ume m (b) Bias risk evaluation results displayed by including studies odel to judge the consistency among the included research results.

The nma. fit () function can perform model fitting and identify potential outliers. The lever plots and DIC values produced by this function can help determine the optimal effect model. The lever diagram presents the comparison between leverageik and Bayesian deviation residuals of all I tests and each of the K arms. This can highlight potential outliers when fitting the model; that is, if the data point is outside the purple arc (x^2^ + y = 3), it might lead to poor model fitting. nma. compare () compares the posterior mean deviation of each data group between consistency and the ume model to judge the consistency among the included research results. Using AE1–2 as an example, the lever diagram and consistency test of its model fitting are presented in Figures 5, 6. In general, the possibility of inconsistency in the included studies was small, and indirect comparisons could be made. It should be noted that because the outcome indicator of this study is adverse reactions, there was a zero value in the data set, and nma. fit () function cannot calculate the leverage ratio, PD, and DIC values of data points. However, it had no impact on subsequent data analyses.

#### 3.4.3 Direct comparison of meta-analysis results

**FIGURE 7:**
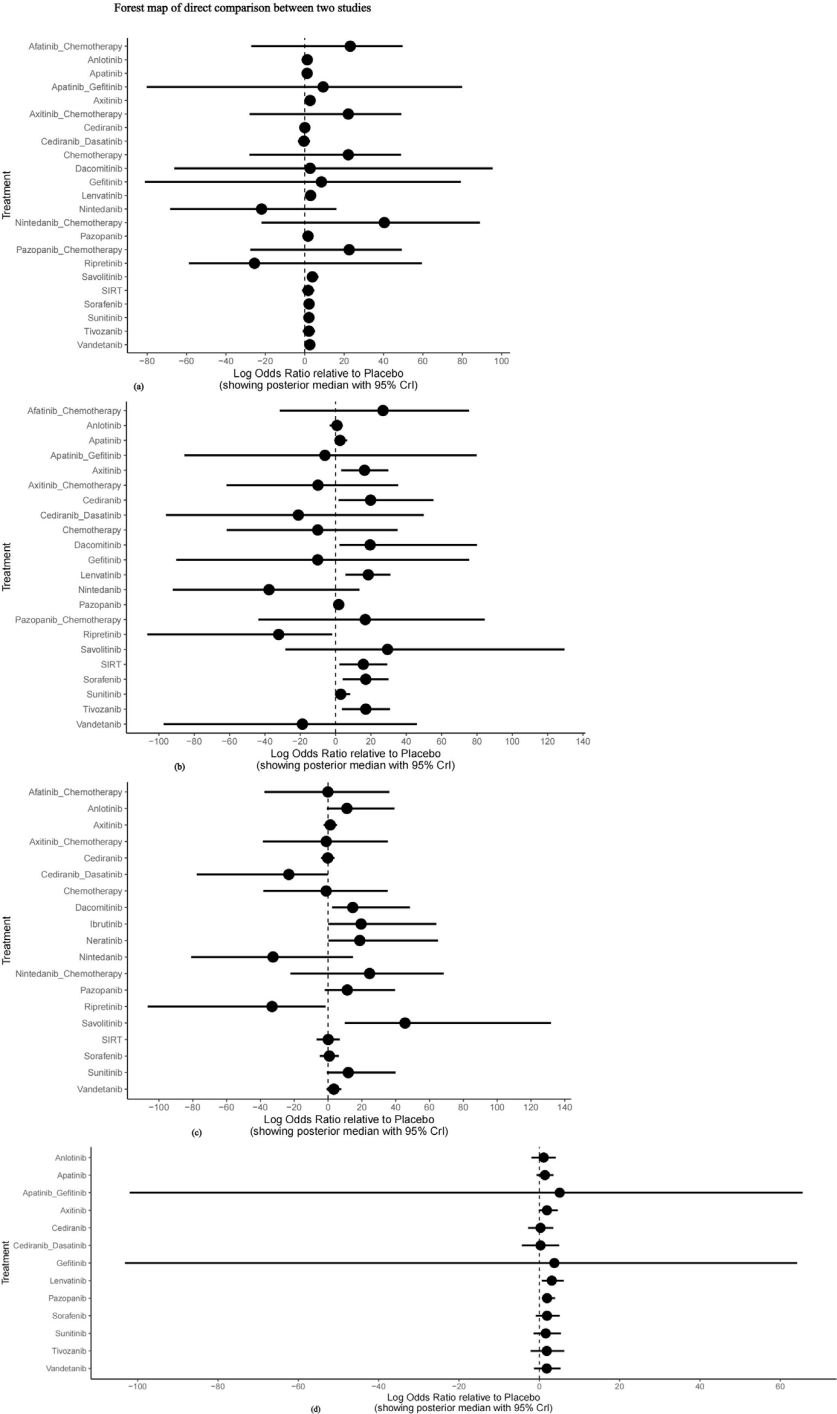
Forest map of direct comparison between two studies. **(A)** Forest map of direct comparison on adverse events (AE) 1–2; **(B)** Forest map of direct comparison on adverse events (AE) 3–4; **(C)** Forest map of direct comparison on elevated serum creatinine levels; **(D)** Forest map of direct comparison on proteinuria.

The nma. forest () function can output forest maps, compare the combined results of drugs in different studies and different effector levels, and present effective intervention measures. Using placebo as the reference, the direct comparison forest map of each outcome indicator is presented in Figure 7. The meta-analysis revealed no significant difference between each intervention group and placebo concerning the incidence of grade 1–2 adverse events, whereas the incidence of grade 1–2 adverse events was higher in the chemotherapy group and TKI combined with chemotherapy group. Axitinib, lenvatinib, sorafenib, and tivozanib had higher risks of grade 3–4 adverse events than placebo. Nintedanib and ripretinib had lower risks of Scr elevation, but the difference between the two groups was not statistically significant. There was no significant difference in the incidence of proteinuria between the intervention groups and placebo.

In conclusion, axitinib, lenvatinib, sorafenib, and tivozanib had higher probabilities of grade 3–4 adverse events than placebo, whereas nintedanib and ripretinib were the safest drugs compared with placebo.

#### 3.4.4 Mesh meta-analysis results

Nma.rank () can compare the posterior probabilities of various intervention measures to achieve the ranking and comparison of NMA analysis results. The SUCRA and rankogram charts intuitively display the sorting probability of each intervention group in the form of curves and histogram. The function parameter larger better is set to FALSE, which means that the risk of outcome indicators increases as the input value increases.

**FIGURE 8:**
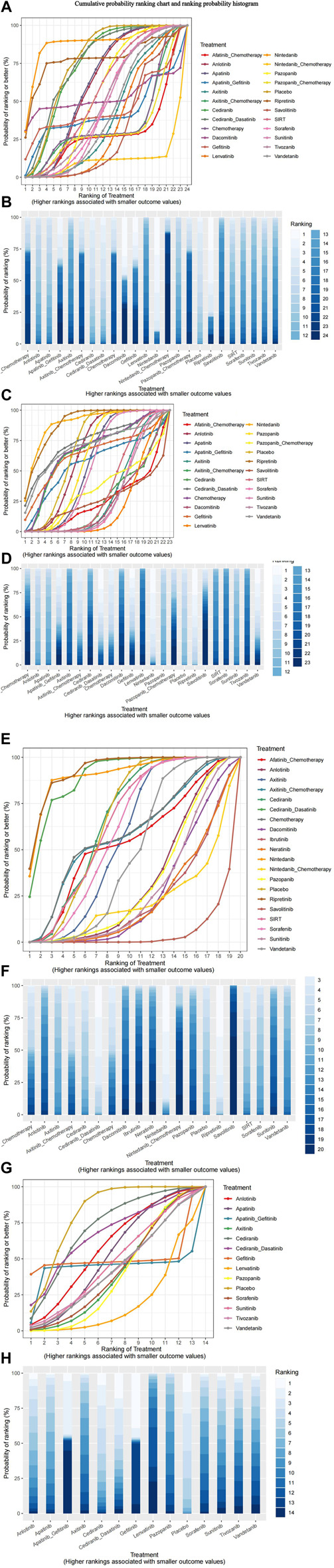
(Continued).

As presented in Figure 8, consistent with the forest plot results, the nintedanib curve was always higher than that of other treatments. This drug had the smallest risk of grade 1–2 adverse events, whereas nintedanib combined with chemotherapy had the highest risk. Ripretinib and nintedanib carried the lowest risks of grade 3–4 adverse events, whereas lenvatinib had the highest risk. Ripretinib, cediranib in combination with dasatinib, and nintedanib had lower risks of Scr elevation, whereas other interventions had higher risks of Scr elevation than placebo. Compared with that for placebo, the probability of proteinuria was higher for all interventions, particularly lenvatinib, gefitinib, and apatinib combined with gefitinib.

The heat map of the ranking table generated by the nma. league () function provided an estimation of the relative effects and permitted comparisons of the relative effects between any pair of interventions. The heat map of each outcome index ranking table is presented in the figure, including the RR and 95% CI of each outcome index in all intervention groups.

**FIGURE 9:**
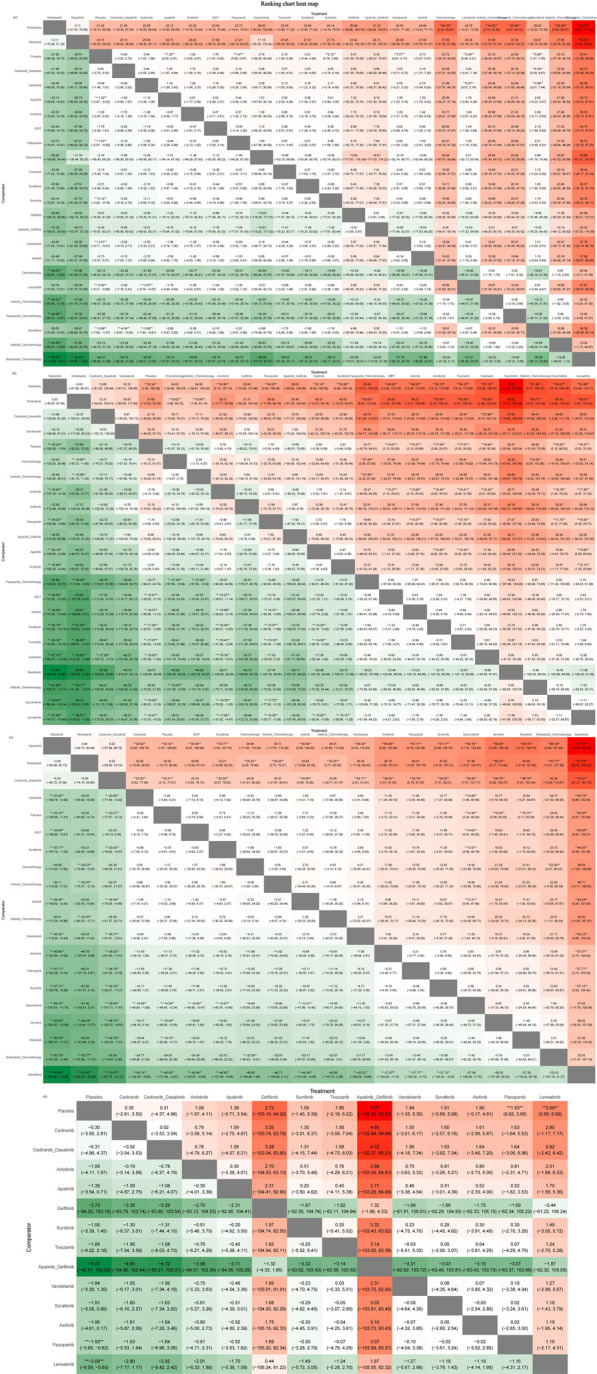
Ranking chart heat map. The heat map of each outcome index ranking table presented comparisons of the relative effects between any pair of interventions, including the RR and 95%CI of each outcome index in all intervention groups. **(A)** Adverse events (AE) 1–2 ranking chart heat map; **(B)** adverse events (AE) 3–4 ranking chart heat map; **(C)** Scr elevation ranking chart heat map; **(D)** proteinuria ranking chart heat map.

As presented in Figure 9, nintedanib and ripretinib carried lower risks of grade 1–2 adverse events than placebo. Conversely, nintedanib combined with chemotherapy, afatinib combined with chemotherapy, and pazopanib combined with chemotherapy had high risks of such events. Whereas nintedanib, ripretinib, and vandetanib were linked to lower risks of grade 3–4 adverse events, afatinib combined with chemotherapy, dacomitinib, and cediranib had high risks of such events.

The interventions with lower risk of Scr elevation were ripretinib, nintedanib, and cediranib combined with dasatinib, whereas those with higher risks were savolitinib and pembrolizumab combined with axitinib. The interventions with lower risks of proteinuria were cediranib and cediranib combined with dasatinib, whereas apatinib combined with gefitinib and gefitinib monotherapy were associated with higher risks.

Among the included studies, four reported data for haematuria. Because of different intervention measures and a lack of evidence, meta-analysis could not be conducted. Therefore, the descriptive report of each research result was as follows (Table 3). In 2017, Yang et alreported the rates of haematuria in patients treated with icotinib (4/85) and WBI combined with chemotherapy (11/73) (Yang et al., 2017). Spreafico et alreported that the incidence of haematuria was lower for cediranib combined with dasatinib (0/11) than for cediranib monotherapy (1/11) (Spreafico et al., 2014). Chi et alstated that anlotinib (30/282) had a higher risk of haematuria than placebo (5/137) (Chi et al., 2021). Zhao et alrevealed that gefitinib combined with apatinib (40/157) had a higher risk of haematuria than gefitinib alone (24/154) (Zhao et al., 2021b).

**TABLE 3:** Studies reporting haematuria results.

Study	Treatment	sampleSize	Bld	Rate
Yang 2017	Icotinib	85	4	4.70%
	WBI_Chemotherapy	73	11	15.07%
Spreafico 2014	Cediranib_Dasatinib	11	0	0
	Cediranib	11	1	9.09%
Chi 2021	Anlotinib	282	30	10.64%
	Placebo	137	5	3.65%
Zhao 2021	Apatinib_Gefitinib	157	40	25.48%
	Gefitinib	154	24	15.58%

## 4 Discussion

Because of their superior selectivity, efficacy, and safety compared with those of traditional chemotherapeutic drugs, some TKIs have become the first-line treatments for cancer in recent years. With the increasing use of these drugs, their renal toxicities are gradually being recognized. Although dose titration is not needed in most conditions and nearly all cases of renal impairment resolve after treatment cessation, therapy discontinuation or a switch to another medicine should be considered in severe cases. Therefore, it is important for clinicians to learn about the risk of kidney injury to select the optimal drug, especially for patients with diminished baseline renal function.

Our study found that renal adverse effects caused by TKIs are common. The highest reported incidence of grade 1–2 adverse events was nearly 70% (Motzer et al., 2013a), and the rate of grade 3–4 adverse events reached a maximum of 19.1% (Zhao et al., 2021b). It should be noted that the nephrotoxicity of TKIs varies greatly among different drugs and studies (the lowest reported incidence is 0%). Scr elevation and proteinuria were the most common events, whereas haematuria was relatively rare. Among the included studies, Scr elevation was recorded by 18 (47.4%) studies, and 23 (60.5%) studies reported proteinuria. Only four (10.5%) studies reported haematuria.

We further ranked the renal adverse reactions of TKIs during cancer treatment *via* network meta-analysis. Overall, TKIs combined with chemotherapy were linked to the highest incidence of renal injury, in which the chemotherapeutic agents played a major role. It has been demonstrated that chemotherapeutic drugs can impair the glomerulus, tubules, interstitium, and renal microvasculature (Malyszko et al., 2017). Nephrotoxicity caused by chemotherapy remains a significant complication limiting the application of these drugs. In terms of treatment with a single drug, savolitinib, dacomitinib, and ibrutinib were associated with greater rates of Scr elevation than other TKIs. Savolitinib is a highly selective mesenchymal epithelial transition factor (MET)–TKI. The underlying mechanism related to renal impairment is unknown. According to a study on the MET–TKI tepotinib, Scr elevation was the most common treatment-related adverse event (63.2%, all grade 1–2) (Sakai et al., 2021). The cause is considered to be reduced creatine secretion by renal tubules resulting from the direct inhibitory effect of the drug on renal tubular transporters. Therefore, the elevation of Scr caused by the reversible interaction with creatinine transporters differs from that caused by true renal failure. Drug adjustment could be unnecessary in this condition, although it is sometimes difficult to define clinically. Dacomitinib is a second-generation irreversible EGFR–TKI that is mainly used to treat non-small cell lung cancer. Because EGFR is also expressed in the kidneys, renal toxicity might be caused by anti-EGFR drugs. EGF is expressed in the ascending portion of Henle’s loop and distal tubules, but the role of EGFR in kidney function remains obscure. Studies on acute kidney injury (AKI) in animal models illustrated EGFR activation could promote renal recovery. However, another study demonstrated that EGFR activation is associated with renal fibrosis in chronic kidney disease (Tang et al., 2013). Recently, a few cases of glomerulonephritis and vasculitis secondary to EGFR–TKI therapy have been reported in the literature (Latcha et al., 2018; Oki et al., 2022), with AKI being most common. Regarding the mechanisms involved, renal hypoperfusion secondary to dehydration is believed to be a prime inducement. EGFR expressed in the gastrointestinal tract is responsible for mucosal integrity and the regulation of ionic transport. Anti-EGFR TKIs can block the negative regulation of chloride secretion and eventually cause diarrhoea (Crosnier et al., 2021). Among the three generations of anti-EGFR TKIs, the second generation, which includes dacomitinib, has the highest incidence of diarrhoea (Rugo et al., 2019). Therefore, AKI caused by these drugs is functional and actually an adverse effect in the context of digestive toxicity. Ibrutinib, which inhibits Bruton TK, is approved for the treatment of chronic lymphocytic leukaemia and mantle cell lymphoma. Scr elevation associated with this agent has been reported in a few previous studies. Dehydration and tumour lysis syndrome have been implicated in the pathogenesis of renal insufficiency (Byrd et al., 2013; Wang et al., 2015). Ripretinib and nintedanib were linked to the lowest incidence of Scr elevation. Both drugs target multiple TKs such as PDGFR and VEGFR (Dhillon, 2020; Schoffski et al., 2021). The reasons for their lower risks of adverse events are unclear.

Among the TKIs, gefitinib had the highest incidence of proteinuria. Similar to other anti-EGFR TKIs, gefitinib can elicit both proteinuria and Scr elevation during cancer treatment. According to a reported case, gefitinib caused nephrotic proteinuria, which was ascribed to an autoimmune response to the drug (Kumasaka et al., 2004). In another case, minimal change glomerulonephritis, the pathogenesis of which is related to an immune disorder, was proven by renal biopsy in a patient with proteinuria after gefitinib treatment (Maruyama et al., 2015). Lenvatinib and axitinib were associated with the second and third highest rates of proteinuria, respectively. Both drugs target VEGFR. Compared with other target therapies, proteinuria appears to be a common side effect on vascular endothelial growth factor (VEGF)-inhibiting agents (Gurevich and Perazella, 2009). The kidneys are rich in VEGF and VEGFR, and their interaction is critical for the maintenance of normal function and integrity of the glomerular basement membrane of kidneys. Thus, the kidneys are highly susceptible to the adverse effects of anti-VEGF medications. Both proteinuria and acute renal failure can occur in patients using these drugs (Izzedine et al., 2014). According to previous studies, the underlying pathologic changes involved proliferative glomerulopathies, thrombotic microangiopathy, and interstitial nephritis (Abbas et al., 2015). Interestingly, cediranib which is also an inhibitor of VEGFR tyrosine kinases showed the lowest risk of proteinuria. Haematuria related to TKIs has rarely been reported. In our study, only four RCTs recorded the incidence of haematuria. In patients treated with anlotinib or gefitinib alone, the rate of haematuria was approximately 10%. It is worth noting that the difference in the incidence of haematuria between the groups in the study was similar to that of proteinuria. This result indicated that haematuria arises from the same mechanism as proteinuria.

In conclusion, our present work provided insights into the previously unreported relative risks of nephrotoxicity associated with TKIs, and nearly all types of TKIs were covered. According to the results, all types of TKIs carry a risk of kidney injury although the underlying mechanisms may be different. Although direct injury, secondary autoimmune disorder, and dehydration have been regarded to contribute to kidney injury, the exact mechanisms remain to be clarified. The infeasibility of performing renal biopsy in patients with tumours in many cases has become the greatest obstacle to exploring this problem. Early studies have suggested that the occurrence of kidney injury depends both on the drug class and agent (Piscitani et al., 2020). Based on their lowest risks of renal adverse events, ripretinib and nintedanib are recommended for the treatment of kidney disease patients with advanced gastrointestinal stromal tumour and non-small cell lung cancer respectively. Cediranib can serve as the first choice in anti-VEGFR treatment when proteinuria is concomitant.

Our study had some limitations. First, some RCTs included in this study did not clarify the used randomization method, and blinding was not used or reported in some studies, which may have led to selection and performance bias. Second, the baseline characteristics of the patients included in the different RCTs, especially the tumour type and treatment strategies, were inconsistent, which may lead to heterogeneity among the studies. For example, patients with renal cell cancer were at higher risk of renal failure because of previous partial or radical nephrectomy (Launay-Vacher et al., 2011). Third, the severity of renal effects might be greatly affected by the different targets of TKIs, which is determined by underlying mechanisms that remain to be elucidated. However, because of the limited data, it is difficult to conduct further subgroup analysis at present. More evidence provided by high-quality studies is needed for future research.

## Data Availability

The original contributions presented in the study are included in the article/supplementary material, further inquiries can be directed to the corresponding author.
